# Management of hydrocephalus associated with large vestibular schwannomas

**DOI:** 10.1016/j.bas.2025.104318

**Published:** 2025-07-03

**Authors:** Leslie Lemnos, Lucas Troude, Mohamed Boucekine, Stéphane Gargula, Anne Balossier, Jean Régis, Pierre-Hugues Roche

**Affiliations:** a Department of Neurosurgery, North University Hospital, APHM-AMU Chemin des Bourrely, 13015 Marseille, France; b Department of statistical analysis, University of medical and paramedical sciences, Aix-Marseille University (AMU), 27 boulevard Jean Moulin, 13385 Marseille, France; c Department of ENT, Conception University Hospital, APHM-AMU 147 boulevard Baille 13005 Marseille, France; d Department of Neurosurgery, Timone University Hospital, APHM-AMU, 264 Rue Saint-Pierre, 13385 Marseille, France

**Keywords:** CSF shunting, Gamma knife radiosurgery, Hydrocephalus, Vestibular schwannoma

## Abstract

**Introduction:**

Some patients with vestibular schwannoma (VS) may present with hydrocephalus.

**Research question:**

In such cases, some authors suggest cerebrospinal fluid (CSF) shunting, while others prefer tumor removal from the outset.

**Material and methods:**

In our study, we retrospectively compared patients for whom we chose to treat the hydrocephalus with CSF shunting and those for whom VS surgery was performed first.

**Results:**

Among a group of n consecutive patients harboring a stage 3 and 4 VS and eligible for resection, 34 patients presented with hydrocephalus. Thirteen patients underwent CSF shunting (group 1). Twenty-one patients had their VS removed first (group 2). Among the latter group, 18 patients had resolution of hydrocephalus. There was a significant difference between group 1 and 2 in the presence of signs of intracranial hypertension (p = 0.00), preoperative tumor volume (p = 0.04).

Previous radiosurgery and a strong adherence of the tumor capsule to the brain were statistically associated with requirement of CSF shunting (p = 0.01).

**Discussion and conclusion:**

The results of this study suggest that VS patients presenting with a well-tolerated hydrocephalus should be preferentially treated of their schwannoma with rare need for a shunt.

## Highlights

• Vestibular schwannomas may present with hydrocephalus.
•Treatment may involve cerebrospinal fluid shunting or tumor resection.
•Previous radiosurgery may associate with requirement of CSF shunting.

## 1 Introduction

Vestibular schwannoma (VS) is benign tumor that develops in the Schwann sheath, with the main symptom hearing loss. In rare cases, approximately 10 per 100 000 people according some authors, a ventricular dilatation may be present, most frequently in the elderly (Marinelli et al., 2019; Tanaka et al., 2003). The incidence of hydrocephalus varies between 4 and 24 % in the literature (Al et al., 2013).

Several mechanisms such as disorders of CSF resorption by excessive level of proteins in CSF or by tumor microbleeding, mass effect on 4th ventricle, have been suggested but without certainty (Al et al., 2013; Hayes et al.). In rare cases, the symptoms associated with hydrocephalus are major, necessitating CSF shunting as a priority. Conversely the hydrocephalus is well tolerated in the majority of patients. This raises the question of how to manage hydrocephalus.

Depending on the team's habits and clinical impact, management of VS associated hydrocephalus varies: some may undergo a CSF shunting (ventriculoperitoneal shunt or endoscopic third ventriculostomy) before tumor removal. Others recommend intraoperative temporary CSF external drainage to facilitate surgical approach to the cerebellopontine angle, cerebral retraction and tumor dissection (Pirouzmand and Tator, 2001). Some authors suggest VS resection may be performed, considering that this would restore CSF circulation and/or normalize the level of proteins in the CSF. CSF shunting is a surgical procedure associated with a certain percentage of complications such as hematoma or infection of surgical site. The indication must therefore be relevant (Schroeder et al., 2002; Martínez-Lage et al., 2015).

The aim of this study was to compare patients harboring a vestibular schwannoma stage 3 and 4 according Koos associated with hydrocephalus treated by a CSF shunting and those treated by VS resection first and identified factors to predict hydrocephalus persistence.

## 2 Methods

### 2.1 Study design

**Fig. 1:**
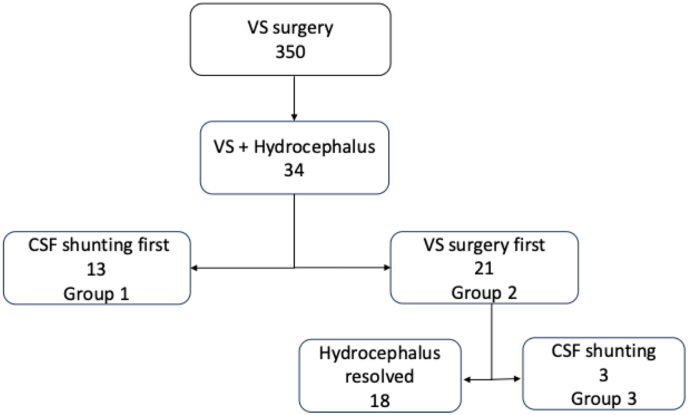
Flowchart patients' study (VS vestibular schwannoma, CSF cerebrospinal fluid).

All patients who underwent a VS resection in North Hospital Marseille between 1998 and 2023 were retrospectively reviewed including the ones presenting with clinical or radiological hydrocephalus. The neurofibromatosis type 2 patients were excluded from analysis. Three hundred and fifty patients underwent a VS resection during this period (Fig. 1). The overall prevalence of hydrocephalus associated with VS was 10 % in our series (34 patients). The diagnosis of VS was histologically confirmed in all cases. Informed consent of all patients was obtained, and our institutional local ethical committee approved this study (Aix-Marseille University Ethics Committee - Authorization number: 2018-24-01-003).

### 2.2 Patients’ characteristics

The following baseline data were collected: age, gender, hearing loss, dizziness, tinnitus, hypoesthesia in trigeminal distribution, cerebellar syndrome and ataxia, hydrocephalus, signs of intracranial hypertension, gamma knife failure and radiotherapy failure.

Facial nerve function was defined according to the House and Brackmann classification (HB) (House and Brackmann, 1985). Hearing was defined according to Gardner-Robertson hearing (Gardner and Robertson, 1988) scale. Radiological examinations (including pre- and postoperative volumetric measurements performed on axial Gadolinium-enhanced T1 weighted MR images– iPlan 3.0 Cranial *Brainlab*, Munich, Germany) were recorded at presentation and during follow-up (Van De et al., 2009). Hydrocephalus was defined as Evans index ≥0.25 on MRI regardless the clinical presentation (Evans, 1942). Surgical findings such as tumor consistency and vascularization, adhesion to cerebellum and brainstem were registered.

### 2.3 Hydrocephalus management

We defined 3 groups of patients: Group 1 included patients with hydrocephalus and signs of elevated intracranial pressure. They underwent a CSF shunting prior to VS resection (ventriculoperitoneal shunt or endoscopic third ventriculostomy). Group 2 included patients with moderate and clinically well tolerated hydrocephalus treated by VS resection first. Group 3 included patient from group 2 who required CSF shunting after VS resection due to persistent balance disorders.

### 2.4 Surgical protocol

The patient could be either operated on through a retrosigmoid or translabyrinthine approach. Under microscope, CSF depletion for brain relaxation was obtained by opening the cerebellomedullary cistern. Then the tumor capsule was opened and the surgeon alternated between intracapsular debulking and peripheral dissection. A maximum safe resection was carried out while preserving the cranial nerves. The intraoperative decision to interrupt tumor resection was driven by deterioration of the electromyography responses during stimulation and/or evidence of critical adherence of the tumor capsule to the facial nerve.

### 2.5 Postoperative outcome

Clinico-radiological follow-up was planned at 6 and 12 months after resection, and subsequently at 2, 3, 5, 7 & 10 years after surgery, and then once every 3 years thereafter. Extent of resection was assessed by the volume of residue on the first a Gadolinium-enhanced MRI 6 months after surgery. Gross total resection (GTR) was defined as 100 % tumor clearance, near total resection (NTR) as 95–99 % tumor removal, subtotal resection (STR) was defined as 80–95 % tumor removal, and partial resection (PR) was defined as < 80 % tumor removal.

### 2.6 Statistical analysis

A comparative analysis of demographics and clinical characteristics was performed for the three groups previously defined. Continuous variables were expressed as means with range or median with Interquartile range. A Stutent's *t*-test or 2-sample Wilcoxon test was performed to assess the differences between groups. Categorical variables were expressed as frequency and percentage and compared using the chi-squared test or Fisher's exact test. Ninety-five percent confidence intervals (CIs) are presented, where appropriate. Following convention, the alpha level of significance was set at 5 %, hence values where p ≤ 0.05 have been referred to as ‘‘significant’’ and those where 0.05≤p ≤ 0.10 as a ‘‘trend’’ or ‘‘marginally significant’’. The statistical software R version 4.4.0 was used in the analysis.

## 3 Results

### 3.1 Population study (Table 1)

The overall prevalence of hydrocephalus associated with VS was 10 % (34 patients). Among hydrocephalus patients: the mean age was 59.5 years (range 21–83) and male-to-female ratio was 12:22. The overall rate of ventricular shunt was 5 %. All hydrocephalus patients presented with hearing loss and balance disorders. The severity of hearing loss at diagnosis was G&R grade 2, 3, 4 and 5 in 12 %, 27 %, 29 % and 32 % respectively. Preoperatively, facial nerve status was HB grade 1, 2, 3, 4 and 5 in 82 %, 9 %, 6 %, 0 %, and 3 % respectively.

**Fig. 2:**
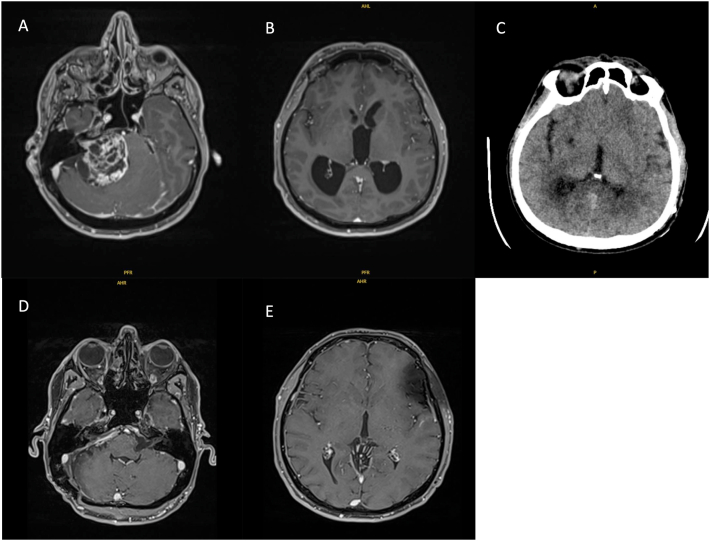
Axial T1-weighted magnetic resonance imaging (MRI) showing (A) a 5,3 cm gadolinium-enhancing tumor within the right cerebellopontine angle (CPA) and (B) hydrocephalus. (C) Axial CT scan after ventriculoperitoneal shunt to treat hydrocephalus. (D) A post operative axial MRI confirmed total removal of tumor and (E) regression of hydrocephalus one year later.

In group 1, 13 patients (38 %), including 9 females and 4 males presented an acute hydrocephalus with headache, severe balance disorders requiring emergency ventriculoperitoneal shunt before tumoral resection (Fig. 2). Their mean age was 63 years (range 34–78). The average tumor volume was 16.37 cc. The mean tumor diameter was 30.9 mm (15–53). All patients had a KOOS 4 tumor.

**Fig. 3:**
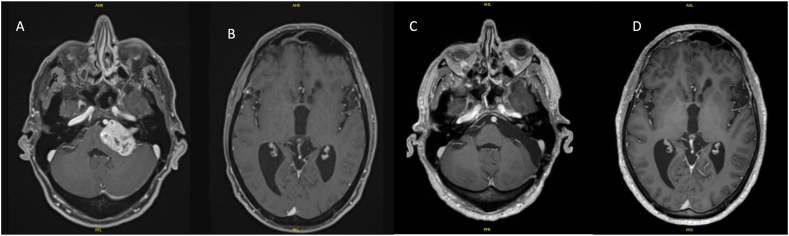
(A) Axial T1-weighted MRI showing a 3,5 cm left CPA tumor with (B) hydrocephalus. (C) A postoperative T1-weighted MRI with gadolinium confirmed total removal of tumor and (D) regression of hydrocephalus one year later.

In group 2, 21 patients (62 %) underwent VS resection as a first line treatment. Their mean age was 57 years (range 21–83) and a male-to-female ratio of 8:13. The average tumor volume was 27.85 cc. The mean tumor diameter was 35.7 mm. There were 2 patients with a KOOS 3 tumor. For 86 % of these patients, tumor resection allowed the resolution of the hydrocephalus without any need for a shunt (Fig. 3). There was a significant difference between group 1 and group 2 in respect of symptomatic intracranial hypertension (p = 0.00) and preoperative tumor volume (p = 0.04). The Evans index was 0.32 in group 1 whereas 0.33 in group 2 (p = 0.6). No difference in the amount of cranial nerves dysfunction could be identified between group 1 and 2.

**Fig. 4:**
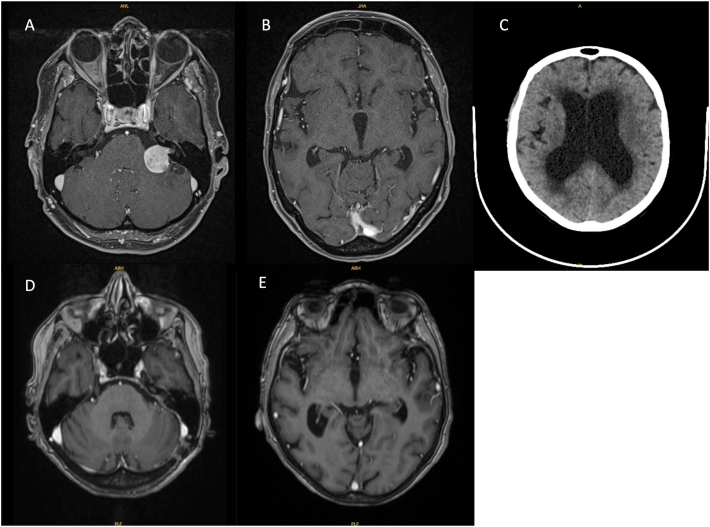
(A) Axial T1-weighted MRI showing a 2 cm left CPA tumor with (B) hydrocephalus. (C) Axial CT scan showing increased hydrocephalus at 48 h postoperatively treated by endoscopic third ventriculostomy. (D) Axial T1 weighted MRI showing total removal tumor and (E) regression of hydrocephalus.

Within group 2, three patients (14 %) (patients 11, 22 and 29) required a CSF shunting several months after surgery (Group 3). Their mean age was 51 years. They presented persistent signs of hydrocephalus including balance disorders despite tumor removal. One patient aged 70 (patient 29) required an endoscopic third ventriculostomy within 15 days. On systematic postoperative CT scan, she had a well-tolerated surgical site hematoma that did not require revision surgery. In the days following, she presented signs of acute hydrocephalus with consciousness disorders. The CT scan showed increasing size of the ventricles as compared to the preoperative scan (Fig. 4). Patient 13 required a CSF shunting 4 months after surgery because of disabling balance disorders and persistent ventricular dilatation. Patient 22 (78 years) required a CSF shunting 10 months after surgery because of Hakim's triad. Anatomopathological analysis of these 3 tumours revealed no nuclear atypia neither elevated mitotic activity.

Postoperatively, the hearing loss worsened with G&R 4 in 3 % and 5 in 97 %. The immediate postoperative facial function was HB grade 1, 2, 3, 4 and 5 in 41 %, 24 %, 6 %, 21 %, and 9 % respectively. At last follow-up examination, 74 % of the patients retained a good (53 % HB grade 1 and 21 % HB grade 2), 21 % a moderate HB grade 3, and 6 % a poor facial function (3 % HB grade 4 and 3 % HB grade 5).

### 3.2 Postoperative complications

Twelve patients (35 %) presented at least one complication. Some complications occurred such as ophthalmic keratitis (12 %), radiological postoperative hematoma which did not require a salvage surgical management (6 %), abducens nerve deficit (9 %), CSF leakage (6 %) resolved medically with two lumbar punctures, long tract sign (6 %), lower limbs deep vein thrombosis (6 %), meningitidis (3 %), consciousness disorders (3 %). There was no significant difference between group 1 and 2 in terms of complications encountered (p > 0.14). Table 1.

**Table 1:** Clinical baseline.

Characteristic	Overall population (%)	CSF shunting first Group 1 (%)	VS surgery first Group 2 (%)	*P*
M/F (%)	12 (35 %)/22 (65 %)	4 (31 %)/9 (69 %)	8 (38 %)/13 (62 %)	*0.7*
Mean age in years	59.5	63	57	*0.2*
Failed radiotherapy	1 (2.9 %)	0	1 (5 %)	*>0.9*
Failed GK	4 (12 %)	4 (31 %)	0	***0.015***
**Clinical features (%)**				
Tinnitus	5 (15 %)	2 (15 %)	3 (14 %)	*>0.9*
Dizziness	7 (21 %)	2 (15 %)	5 (24 %)	*0.7*
Instability	26 (77 %)	10 (77 %)	16 (76 %)	*>0.9*
Cerebellar syndrome	22 (65 %)	8 (62 %)	14 (67 %)	*>0.9*
Long tract signs	5 (15 %)	2 (15 %)	3 (14 %)	*>0.9*
High intracranial pressure	21 (62 %)	12 (92 %)	9 (43 %)	***0.005***
**Radiological features**				
Location Left/Right	16/18	4 (31 %)/9 (69 %)	12 (57 %)/9 (43 %)	*0.13*
Mean tumor diameter (mm)	33.3	30.92	35.71	*0.062*
Mean tumor volume (cc)	22.1	16.37	27.85	***0.040***
Mean Evans index	0,33	0.32	0.33	*0.6*
**Complications**				
Keratitis	3 (9 %)	1 (7.7 %)	2 (9.5 %)	*>0.9*
Corneal ulcerations	1 (3 %)	0	1 (4,8 %)	*>0.9*
CPA hematoma	2 (6 %)	0	2 (9.5 %)	*0.5*
Abdominal hematoma	0	0	0	
Wound infection	0	0	0	
Meningitidis	1 (3 %)	1 (7.7 %)	0	*0.4*
CSF leakage	2 (6 %)	0	2 (9.5 %)	*0.5*
Subcutaneous collection	1 (3 %)	0	0	
Paresis VI	3 (9 %)	2 (15 %)	1 (4.8 %)	*0.5*
Long tract signs	2 (6 %)	2 (15 %)	0	*0.14*
Decubitus complications	2 (6 %)	1 (7.7 %)	1 (4.8 %)	*0.9*
Coma	1 (3 %)	1 (7.7 %)	0	*0.4*

### 3.3 Predictive factors of CSF-shunt required

In our series of patients with 316 schwannomas without hydrocephalus, which we can refer to as “group 0”, 26 patients reported history of preoperative GKRS, i.e 8 %. In our series of patients, radiosurgery appears to be statistically associated with an increased risk of CSF shunting, even following tumor resection. (Table 2). Among 34 patients with hydrocephalus, 4 (12 %) patients had been previously treated with GKRS. All these patients required a CSF shunting before schwannoma resection (p = 0.00). In addition, a post-gamma knife arachnoiditis was an intraoperative finding observed in 100 % of cases, as observed in the patients from the “group 0” (Troude et al.). A strong adherence of the tumor capsule to the cerebellum and/or middle cerebellar peduncle was statistically associated with requirement of CSF shunting (67 % vs 23 % respectively - p = 0.01).

**Table 2:** Surgical field.

Surgical data	Overall population (%)	CSF shunting first Group 1 (%)	VS surgery first Group 2 (%)	*p*
Arachnoiditis post GK	5 (15 %)	5 (38 %)	0	***0.005***
Brainstem adhesion	7 (21 %)	2 (15 %)	5 (24 %)	*0.7*
Cerebellar adhesion	17 (50 %)	3 (23 %)	14 (67 %)	***0.013***
Residual volume				*0.8*
0	1 (3 %)	0	1 (5 %)	
≤1 cc	22 (56 %)	7 (54 %)	12 (57 %)	
1<x < 2 cc	5 (15 %)	3 (23 %)	2 (10 %)	
>2 cc	9 (26 %)	3 (23 %)	6 (29 %)	

## 4 Discussion

There was a significant difference between group 1 and group 2, the former one displaying obvious signs of raised intracranial hypertension and leading to CSF shunt first. We can conclude that in these patients, hydrocephalus symptoms predominated, whereas in group 2, the symptoms were more related to the tumor, with well-tolerated hydrocephalus. Our strategy worked rather well, as in 85 % of cases, the initial tumor removal resolved the hydrocephalus, thereby avoiding the potential complications of shunting. In Table 3, we compared data from the literature.

**Table 3:** Literature review

Authors	Study design	Patients with VS	Patients with HC	CSF shunting first	VS surgery first	VS surgery followed by CSF shunting	Only CSF shunting
Fairhead et al., 2023	Retrospective	204	30	20	9	3	8
Shin et al., 2021	Retrospective	NA	128	68	60	NA	NA
Di Russo et al., 2021	Retrospective	NA	290	27	202	20	9
Al Hinai et al., 2013	Retrospective	250	3	1	1	1	1
Miyakoshi et al., 2013	Retrospective	405	57	0	31	0	NA
Tanaka et al., 2003	Retrospective	236	33	9	24	7	NA
Pirouzmand et al., 2001	Retrospective	NA	39	10	23	5	4
							

VS: Vestibular Schwannoma, HC: Hydrocephalus, CSF: Cerebro-Spinal Fluid, NA: No Available.

However, some teams propose other strategies. Some suggest transient CSF drainage (Di Russo et al., 2021). Others propose a grading system to predict the need for CSF shunting following posterior fossa surgery including preoperative hydrocephalus, tumor location and perilesional oedema in case of extraparenchymal tumor fossa (Won et al., 2020). However, only 38 % patients with vestibular schwannoma were included in their study and vestibular schwannoma are usually not associated with perilesional oedema in our current practice (Won et al., 2020).

In retrospective statistical analysis of our data, we found that there were no significant differences in age, gender or Evans index. Paradoxically, we found a significant difference between our two groups in terms of tumor volume. This was smaller in patients who had undergone CSF shunting prior to schwannoma excision. Some authors suggested that hydrocephalus associated with smaller VS is due to high level of protein in CSF, and less tolerated than progressive obstructive hydrocephalus associated with larger VSs (Al et al., 2013; Fukuda et al., 2007; Gardner et al., 1954). Unfortunately, in our study, the concentration of protein in CSF wasn't available excepting for one patient. We also found that patients who had previously undergone radiosurgery were shunted in priority, due to ill tolerated intracranial hypertension. In the literature, several studies have already reported on the consequences of radiosurgery, such as necrotic tumor tissue on CSF resorption mechanisms, or on the thickening of arachnoid membranes (Sawamura et al., 2003; Roche et al., 2008; Miyakoshi et al., 2013; Balossier et al.). An obstructive hydrocephalus by compression of the ventricular pathway may also occur by tumor growth after GKRS failure. This situation is of exceptional occurrence. Actually, it well documented that a large group of VSs show evidence of transient swelling remodeling after GKRS treatment, turning out to be pseudo-progression with delayed tumor control for a vast majority of them on longer follow-up (Troude et al.; Balossier et al.; Carlson et al., 2021; Lee et al., 2016). Nevertheless, none of the patients who experienced this transient swelling (70 % of the VSs treated with GKRS) or treatment failure in our institution required surgery because of hydrocephalus.

Moreover, we did not find any link between the extent of surgical excision and the occurrence of postoperative hydrocephalus. Last, our findings couldn't confirm that evidence of cystic VS could be a predictor for the persistence of post-operative hydrocephalus as reported by reported by Shin et al. (2021).

## 5 Limitations

We present a retrospective cohort study, though patient follow-up was prospectively conducted. In spite of a large number of operated schwannomas, the cohort of patients who underwent CSF shunting is low, particularly in group 3. Measurement of intrathecal protein levels wasn't done systematically. Answering properly the question of shunting or resecting the schwannoma of group 1 patients would require a randomization which isn't acceptable for safety reasons. According to the Evans index, the threshold was initially defined as 0.3 in 1942. Several authors suggest a more appropriate threshold of 0,25 (Brix et al., 2017, Zhou and Xia, 2022).

## 6 Conclusion

In this retrospective analysis of VS patients presenting with hydrocephalus we presented our philosophy for the management of this small sample. We considered that a shunt was of utmost priority in cases of ill-tolerated intracranial pressure, while we offered VS resection for the other patients with a need to put a shunt in only 14 % of these patients. Further studies are needed in order to predict which patients will require shunting in this very confidential group of patients.

## Consent to participate

Informed consent was obtained from all patients.

## Availability of data and material

My manuscript has data included as electronic supplementary material.

## Code availability

Not applicable.

## Ethics approval

Comité d’Ethique de l’Université d’Aix-Marseille (Authorization number: 2018-24-01-003).

## Consent for publication

All patients agreed for publication.

## Submission statement

This manuscript has not been previously published in whole or in part or submitted elsewhere for review.

## Author contributions

All authors have made substantial contributions so as to qualify for authorship, and have read and approved the final version of this manuscript.

LT, JR, PHR: conception and design of the study, LL, LT, AB: acquisition of data & drafting the Article,LL, MB, LT: analysis and interpretation of data,JR, PHR: critically revising the Article.

## Disclosure of funding

None of the authors disclose any financial disclosure in relation with this study.

## Conflict of interest

None of the authors disclose any conflict of interest in relation with this study.
